# Supplementary material for: Deep coral habitats of Glacier Bay National Park and Preserve, Alaska
Source: PLoS One. 2020 Aug 4;15(8):e0236945. doi: 10.1371/journal.pone.0236945 (PMC7402505; doi:10.1371/journal.pone.0236945)
Supplement: S3 Fig — (a) The species accumulation curve, S observed, shows the average observed species for each site in the Far zone (C1, C2) with standard deviation error bars present. (b) Average CHAO 1 richness estimator curves for each site in the Far zone (C1, C2) with standard deviation error bars present. (DOCX) [file pone.0236945.s003.docx]

(a)

(b)

**S3 Fig**. Species Accumulation Curves for Sites in Far Zones. (a) The species accumulation curve, *S* observed, shows the average observed species for each site in the Far zone (C1, C2) with standard deviation error bars present. (b) Average CHAO 1 richness estimator curves for each site in the Far zone (C1, C2) with standard deviation error bars present.
