# Supplementary figures and images for: Deep coral habitats of Glacier Bay National Park and Preserve, Alaska
Source: PLoS One. 2020 Aug 4;15(8):e0236945. doi: 10.1371/journal.pone.0236945 (PMC7402505; doi:10.1371/journal.pone.0236945)

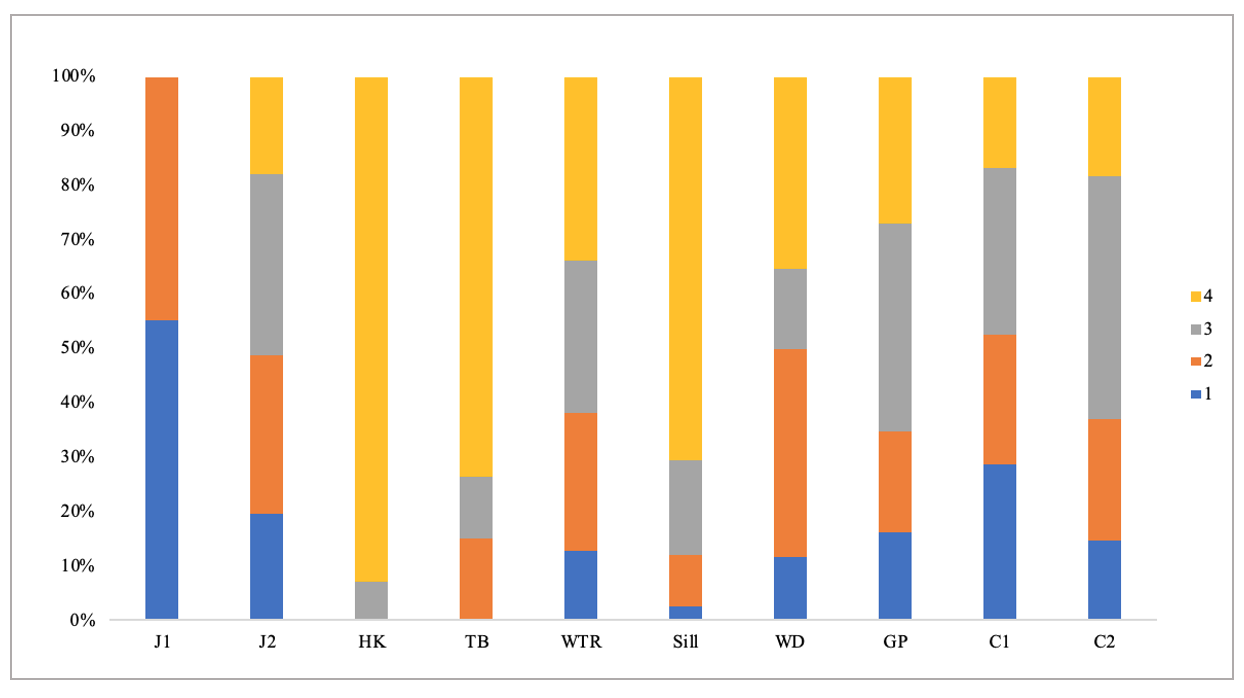


**S4 Fig**. Frequency of *Primnoa pacifica* size class occurrence at each site.

Supplement: S4 Fig — (DOCX) [file pone.0236945.s004.docx]
