# Supplementary material for: Deep coral habitats of Glacier Bay National Park and Preserve, Alaska
Source: PLoS One. 2020 Aug 4;15(8):e0236945. doi: 10.1371/journal.pone.0236945 (PMC7402505; doi:10.1371/journal.pone.0236945)
Supplement: S1 Table — (DOCX) [file pone.0236945.s005.docx]

**S1 Table. Taxon Contribution to Similarity Between Sites – SIMPER Analysis.**

|  | **Species Contribution to Similarity between Zones (%)** | | | **Species Contribution to Similarity within Zones (%)** | | |
| --- | --- | --- | --- | --- | --- | --- |
| **Species** | **Near & Mid Sites** | **Near & Far Sites** | **Mid & Far Sites** | **Near Sites** | **Mid Sites** | **Far Sites** |
| Brachiopod | 92.1 | 88.44 | 87.24 | 21.89 | 25.44 | 12.31 |
| *Primnoa pacifica* | 85.49 | 93.65 | 89.94 | 21.84 | 31.28 | 22.27 |
| Crimson Anemone | 94.42 |  |  | 16.47 | 9.98 | 8.7 |
| Hydrozoa | 75.11 | 82.42 | 82.19 | 13.88 |  | 11.39 |
| Stoloniferan coral | 88.4 | 91.4 | 92.06 | 12.03 | 8.38 | 10.41 |
| Porifera | 94.07 | 94.74 | 95.72 | 7.72 | 10.62 | 4.98 |
| Giant barnacle | 81.64 | 85.21 | 87.14 |  | 7.36 | 8.59 |
| Feather duster worm | 95.58 |  |  |  |  |  |
| Solitary cup coral |  | 91.09 | 90.58 |  |  | 5.97 |
| Brittle star |  | 91.61 | 90.69 |  |  | 6.72 |
| Nudibranch |  | 94.47 | 95.67 |  |  |  |
| Triton snail | | 94.65 | 96 |  |  |  |
| **Average Overall Similarity** | 66.84% | 65.09% | 68.92% | 70.63% | 78.64% | 72.31% |
|  |  |  |  |  |  |  |
