# Supplementary material for: Deep coral habitats of Glacier Bay National Park and Preserve, Alaska
Source: PLoS One. 2020 Aug 4;15(8):e0236945. doi: 10.1371/journal.pone.0236945 (PMC7402505; doi:10.1371/journal.pone.0236945)
Supplement: Attachment — Submitted filename: Review of PONE-D-20-05571.pdf [file pone.0236945.s006.pdf]

REVIEW

Deep benthic coral habitats of Glacier Bay National Park and Preserve, Alaska

Hartill et al.

General comments
The manuscript is generally well-written, but sentences are sometimes unnecessarily cumbersome. I have suggested some edits that may help clarify and streamline the manuscript.

The authors use a mix of deep-sea and cold-water corals in describing their focal community. I recommend picking a term and using it consistently. Since the focus of the paper is a high latitude, relatively shallow ecosystem, I would use CWC instead of deep-sea.

This work is worthy of publication – it focuses on important benthic habitats that provide Essential Fish Habitat and support keystone species. The study is focused on a high latitude ecosystem that is susceptible to climate change, and it is valuable to understand current species' distributions from which to measure future change. However, I have a number of comments and concerns that I think need to be addressed before this is suitable for publication. I therefore recommend publication with major revisions.

Title
Benthic doesn't seem necessary

Abstract
L1: Suggest changing to '…southeastern Alaska, comprising a system of fjords….

Keywords
I suggest adding cold-water corals instead of, or in addition to, diversity

Introduction
L28: while this statement is still true, the authors should acknowledge that there have been many CWC studies conducted, particularly over the past decade. Freiwald and Roberts is quite out of date now. Amend statement and add new references.

L30: Add comma after 'foundation for,' and 'that sustain,'

L33: Move 'in areas such as ……Scandanavia' to L43, replacing 'at these four locations'

L34: Add 'than usual' after 'shallower depths'

L39: Explain why a freshwater layer would result in reduced light. I also suggest replacing 'enhanced darkening' with 'reduced light'

L47: Delete 'Among these high latitude fjord systems where'

L49: Suggest adding (commonly known as the Red Tree Coral) after Primnoa pacifica

L50: Replace ‘deep-water’ with ‘deeper’ – 20 m cannot be considered deep-water

L55: Replace ‘is’ with ‘encompasses’ after GBNPP

L65: Delete ‘scale’.
L69: Delete ‘commercial fishing activity and other’ and add a phrase or sentence that acknowledges climate change as a major anthropological factor
L74-84: This section describes the area in detail, but there is no frame of reference since these places are not marked on the map. The authors should add labels to Fig. 1, showing some of the more important features described in this section. The map also needs study site labels.

L88: I assume ‘vegetation’ is terrestrial not aquatic, but please clarify

L91-92: I don’t see how sedimentation creates stratification, which is the implication of this sentence. I suggest removing ‘due to increased sedimentation’ and starting the next sentence with. ‘Increased sedimentation from run-off leads to lower light…’

L94: Replace ‘shallow ocean’ with ‘photic zone’

L95: Replace ‘related material’ with ‘other organic’

L99: Explain why optimal conditions occur ‘where fjord and shallow sill processes meet’

L100: Do these statements apply to all/most fjords or are they specific to the study area? Clarify, and if the latter, provide a depth for the ‘deep central basin’

L109: Suggest deleting ‘dichotomously branching’, which is a specific taxonomic term and should be defined if used.

L112: replace ‘in’ with ‘of’

L115-119: This is a long and rather cumbersome sentence. I suggest re-wording to ‘The large, complex structure of P. pacifica colonies provides habitat for a diverse community of associated species, some of which (such as rockfish and crabs) are economically important (Stone….etc).

L119: Suggest re-wording to ‘Primnoa pacifica therefore exhibits keystone…..’

L122: This is a rather vague and unsubstantiated statement. Explain or delete.

L126-128: The authors reported a study in 2010 that surveyed areas 180 m deep, so this survey is actually isn’t the first deep sea (defined by NOAA as >50 m) study. Suggest re-phrasing to ‘This study expands on earlier surveys in the GBNPP to assess benthic community structure from 100-300 m using a remotely operated vehicle (ROV). **Note –** the 100-300 m depth range is stated by the authors, but table 1 indicates the shallowest depth surveyed was 197 m. Clarify/adjust

Materials and methods

This section could be better organized. Start with site selection, describe the vehicle and associated instruments in full detail, describe survey methods, then statistical analysis and data treatment. Specific comments below.

L136: Delete a priori – not appropriate context.

L137-139: Move to later section on ROV surveys

L139-142: This section says sites were selected based on existing knowledge of coral distribution, but L136 states multibeam was used for site selection – clarify.

L148: Table 1. Site name abbreviations should be added to the map. Error (st. dev) should be included in the temp and salinity averages. Transect depth range should be used instead of start depth as transects move up steep walls. Delete Station # - it has no context. The total transect areas are very small for ROV surveys. Please explain why in the text.

L150: This sentence is rather confusing. Vertical transects imply ascending a wall (maybe the authors mean the camera was oriented vertically?), whereas 'along the seafloor' implies a more horizontal aspect. The phrase 'beginning near the deep central axis then moving to the base of the wall' again implies some horizontal survey prior to the vertical. If this was the case for every dive, there would likely have been a mix of habitat types surveyed, which would have confounded the community analysis.

L156-157: Unclear what this means – how does splitting the video into quadrats compensate for different transect lengths?

L158: 'Portions were selected based on presence of epibenthic fauna' – this sounds like a confounding factor for a community analysis – please explain.

L164: Replace 'A CTD….' with A Sea-Bird SBE-19 data logger (Sea-Bird Electronics Inc.) was mounted to the ROV and collected…… should this be depth not density? Add units.

L169: Rephrase to 'assess the variation' – confirm implies an a priori assumption of uniformity.

L175: Terminology – morphospecies isn't used in the species richness table – or presumably the calculation. Do the authors mean species? Taxa?

L176: Total area surveyed – within a transect? Across transects? 5% seems rather arbitrary – how did the authors decide on this number?

L180: How were size classes estimated? The description says coral cover was assessed in 2 dimensions but it is unclear how this translated to size class.

L189: How was Primer E used to estimate diversity?

L191: which data? Percent cover is mentioned earlier, but if % cover was the only variable used, why/how was the data standardized? Explain why the square root transform was used

L194: One way ANOSIM? Rephrase, '….were conducted to determine whether there were significant differences in community assemblages among sites..' – with distance from glacier as a factor?

Results and Discussion

L203: One would expect steep hard substrate on the walls, but not in the central axis. The authors were targeting the walls so its reasonable to expect this habitat would dominate their observations. Suggest deleting '…as would be expected….'

L215: Incorrect use of morphospecies. Use different term – e.g. taxa.

L220: I suggest using scientific names rather than common names (ophiuroids, holothurians, echinoids, asteroids)

L228: Table 2 doesn't present by site as the table title suggests - species lists are grouped by geographic location. My preference as a reader would be to have all sites listed separately, not combined. It would also be helpful to have the site abbreviations added to the location column titles.

L231: Spell out *Primnoa pacifica* in the figure legend

L239: Clarify whether these species richness values are low/high in the context of other fjords etc (in which case, supply references), or whether the authors are simply using relative terms. If so, use relative terms to describe observations.

L242: Delete 'The data at'. The difficulty is not using an ROV, but that the coral canopy hides the below-canopy substrate. Re-word.

L249-254: This section repeats L241-244. Combine and streamline information.

L257: Either delete '….and species associated….' Or expand. It's currently rather a loose end.

L259-262: While this statement may be valid, it is not an example of depth-related differences – in fact as a general rule food decreases with depth.

L262-264: While this may be true, the context is unclear. There is no apparent depth related species turnover observed between the shallower and deeper sites in this study.

L264-265: Needs reference. Also "restricted' is rather subjective and there are many deep sea species to which this statement does not apply – Pp, being one of them. Again, it's also unclear what the context/argument is here. There doesn't seem to be any evidence in this paper to support

zonation or species turnover by depth. There are larger colonies of Pp in the west arm mid sites, but it is unclear why. There are too few replicates and too many other confounding factors to say with certainty.

L269: Expand on ‘patchiness of benthic community assemblages’ – are the differences associated with underlying geology? Some physical factor such as locally elevated currents? Nothing apparent?

L277: The Michaelis et al 2019 was in the North Sea – clarify the similarities between that project and the current study.

L278: It is unclear which spatial gradients the authors are referring to.

L283-284: These sites have very different underlying geology – wouldn’t this be a primary reason for the differences?

L289-290: The text states that the taxa that contributed to 50% of dissimilarity between C1 and C2 were brachiopods, hydrozoan turf and barnacles. SI Table 1 shows similarity (not dissimilarity) within zones. The table only has species names not common names as in the text. I searched the paper and found the taxonomic name for barnacles and brachiopods but the reader should not have to rummage through the document to find pertinent information. SI Table 1 has a comparison of % similarity within zones, but the figures for barnacles, brachiopods and turf in the far zone columns do not add up to 50%. Something needs to be adjusted/clarified. C1 was also classified as primarily soft sediment, but Figure 4 shows C1 assemblages are dominated by hard substrate fauna.

L302-303. The C2 site was furthest from a glacier as stated in the text; however the distance between C1 and C2 was 1.5 km, so distance does not seem to explain the difference in clustering between these two sites.

L305-307: The MDS plot does not seem to support the statement in the text that the MDS plot ‘suggests patterns of fidelity in species dominance determined not only by their proximity to glaciers but also by which fjords they inhabit’. Sites J1 J2, WTR and SILL are all near glacier sites but are in different clusters. HK, TB and C1 are similar distances from glaciers but do not cluster together. WTR, SILL (east) clusters closer with HK and TB (west) than WD and GP (east). It seems that substrate type may be driving the clustering of C1, WD and GP rather than proximity to glaciers or fjord arm.

L323-324: ‘…support our findings of lower diversity and abundance at the near glacier sites’. While this is true for J1 and J2, sites WTR and SILL have similar species richness to the mid sites HK and TB which are 40 km from the glaciers.

L363: It is unclear what the authors mean by ‘classical succession’. Retreating glaciers exposing hard substrate is not an example of ecological succession, which occurs when pioneering species modify the habitat to create conditions appropriate for the successive species.

Conclusions

L412: This statement as written is not accurate – there have been other studies of Pp communities at depths up to 180m, which is defined as deep-sea.
L415: Not consistently according to the data in this paper.

Figures

Fig 1: add site abbreviations and important features that will help interpret the data. Replace 'studies' with 'study' in caption.

Fig 3: Spell Primnoa pacifica the first instance it occurs in a caption, and P. pacifica thereafter

Fig 7: same comment as above

SI Table 1. Add common names to species list to enable comparison with other tables in text. Why is species contribution to **dissimilarity** between zones and **similarity** within? Is the table meant to be set up this way? If so, explain why in the table caption. The averages on the bottom row are clearly not averages of the columns. Explain why in the table caption.

Fig 1 b: The sites are numbered, not named as in the other graphs.

Fig 2 b: Same comment as above
